# On dedicated institutions and funding to support disaster risk reduction: Reflections on local instrumentation in Chile

**DOI:** 10.4102/jamba.v18i1.1985

**Published:** 2026-08-17

**Authors:** Ricardo Fuentealba, JC Gaillard

**Affiliations:** 1 Institute of Social Sciences, Universidad de O’Higgins, Rancagua, Chile; 2 Center for Interdisciplinary Research on Disaster Risk, Resilience and Recovery (CIGIDEN R+), Santiago, Chile; 3 Te Kura Mātai Taiao, Waipapa Taumata Rau, Aotearoa New Zealand; 4 Unit for Environmental Sciences and Management, North-West University, Potchefstroom, South Africa

**Keywords:** Chile, disaster policy, disaster risk management, disaster risk reduction, instrumentation, municipalities

## Abstract

Stakeholders often justify limited disaster risk reduction (DRR) action by citing insufficient funding and a lack of dedicated legislation and institutional infrastructure. This has changed thanks to the Hyogo and Sendai Frameworks, as national governments have created dedicated DRR ministries or agencies, resulting in widespread policy adoption and funding increases. Evidence shows these institutionalisations can instrumentalise DRR under neoliberal agendas driven by international donors. This article, however, highlights how the fixation on dedicated laws, institutions and funding has produced a siloed DRR policy. Using Chile as a case, and the implementation of the Law 21.364, it describes the instrumentation created to deal with disasters at the local level, including DRR and emergency plans, as well as new functions and municipal structures. Given Chile’s subnational governance, municipalities are forced to self-fund a policy issue that is not prioritised, and it leads to the separation of risk reduction, climate change and development into discrete sectors. This obsession with everything-should-be-dedicated results in a fragmentation that diverts attention from the structural causes of risk creation embedded in neoliberal development.

**Contribution:**

The analysis emphasises the need to rethink dedicated DRR institutionalism towards integrated, adaptive approaches addressing underlying risk drivers rather than isolated sectors.

## Introduction

Stakeholders of disaster risk reduction (DRR), that is, initiatives ‘aimed at preventing new and reducing existing disaster risk’ (United Nations Office for Disaster Risk Reduction [UNDRR] 2017), often explain and justify their inaction or, in better cases, their limited action by the lack of sufficient and specific funding. Furthermore, the scarcity of funding is blamed on the lack of dedicated legislation and institutional infrastructure to address disaster risk. The equation is hence simple: no law nor policy, thus no institution and no funding, therefore no action. To overcome this challenge, the Hyogo Framework for Action (HFA) and eventually, the Sendai Framework for Disaster Risk Reduction (SFDRR), have encouraged national governments to legislate and establish designated ministries or offices to make DRR visible and a national and local priority (United Nations International Strategy for Disaster Reduction [UNISDR] 2007, 2015). As a result, national policies and dedicated agencies have mushroomed, especially at the national and regional government levels, and a large amount of dedicated funding has been pumped into DRR all over the world (UNDRR 2023).

The creation and implementation of such laws, policies and funding schemes, however, is no silver bullet. Analyses have shown that dedicated institutions and specific funding foster actions towards reducing disaster risk that are specific rather than holistic (for example, focused only on an early warning system), and project-based and short-term, depending on targeted funding schemes (Lavell et al. 2025). Moreover, this might contribute to the instrumentalisation of DRR within a neoliberal agenda that encourages government agencies (and non-government organisations) to implement initiatives devised by international organisations, donor agencies and/or private companies (e.g. Cheek & Chmutina 2022; Gaillard 2021; Tierney 2015). Furthermore, international funding schemes directed to dedicated government agencies come with strings as per the injunctions of the International Monetary Fund’s and the World Bank’s structural adjustment programmes. As such, the establishment of designated institutions to promote DRR may be perceived as a means of control over the least powerful states.

This short article focuses on the implementation of DRR in Chile to show another ongoing consequence of well-intentioned disaster policies: critically, the obsession with everything-should-be-dedicated, that is, policy, institution and funding, has led to the creation of silos in disaster policy. As we show, with the implementation of a new disaster law, dedicated institutions, tools and funding schemes are turning DRR into another sectoral policy. This results in the differentiation of having some tools and agencies devoted to reducing risks, others to tackle climate change and its effects, and yet others to deal with land uses and development (Núñez 2023). Given the prominent neoliberal character of Chilean disaster policies (Carraro, Visconti & Inzunza 2021; Saavedra, Azócar de la Cruz & Fernández-Vicente 2021), also prevalent in other countries and regions, this siloed policy implementation moves attention away from the structural processes that lead to the creation of disaster risk in the first place (Bankoff & Hillhorst 2022; Wisner et al. 2025).

We analyse Chilean disaster policy from an instrumentation perspective, that is, understanding this policy as a condensed form of knowledge that is not neutral but actively produced through a set of devices (Lascoumes & Le Gales 2007). Lascoumes and Le Gales (2007:9) argue that ‘… instrumentation is really a political issue, as the choice of instrument – which, moreover, may form the object of political conflicts – will partly structure the process and its results’. Similarly, as argued by James C. Scott (1998), the state creates particular ‘projects of legibility’ that turn into practices of measurement and ways of seeing: the state and its institutions produce certain maps, tools, procedures, files, actions, programmes and so on. Thus, we focus on the Chilean disaster policy to describe how it has been implemented at the local level and how it is producing an ineffective DRR silo.

Up next, we describe the emergence of the new policy in a post-disaster context, particularly in the aftermath of the 2010 earthquake and tsunami, given its role in producing an institutional crisis. Then, we focus on the subnational arrangements and tools created by this law, including the development of plans and municipal structures. We end by discussing some shortcomings of the siloed DRR.

## The genesis and aims of Chilean disaster risk reduction policy

Despite the historical advancement of seismic norms and other disaster policies in Chile (Camus et al. 2016), the 8.8 MW earthquake and associated tsunami of 2010, on the 27 of February (27F hereafter), pushed forth myriad transformations. Its occurrence represented a profound institutional crisis in Chilean disaster policy (Cuevas & Flores 2020; Vergara-Saavedra & Ejsmentewicz 2024). The earthquake, and especially the tsunami, exposed a lack of coordination between technical State agencies that were responsible for monitoring seismic and tsunami threats and civil protection; as argued by Farías (2014), Chilean agencies *misrecognised* the tsunami as a threat due to different information and tensions between knowledges. This led to a widespread questioning of public policy and its institutions regarding their capabilities for emergency prevention and response, mainly as the expression of a traditionally reactive approach that characterised the then National Emergency Office (ONEMI [*Oficina Nacional de Emergencia*]). This office followed a policy paradigm focused on top-down, reactive response and short-term rehabilitation of the affected areas, with scant resources to implement plans and activities to mitigate and prevent risks (Cuevas & Flores 2020). Given the extent of social and political damage, and in the face of failures in the existing instrumentation, the 27F represents an institutional crisis that led to a transformation of disaster policy (Nohrstedt 2022).

Eventually, and after more than 10 years of parliamentary debate, the Law 21.364 was enacted in 2021 (Biblioteca del Congreso Nacional de Chile [BCN] 2021). This law establishes a new normative framework to manage disaster risks, moving away from the emergency-centred ONEMI. It also created the new *Servicio Nacional de Prevención y Respuesta ante Desastres* (National Service for Disaster Prevention and Response, SENAPRED). The law redefined the roles and responsibilities of all state actors, emphasising inter-institutional and public–private coordination and enhancing subnational action at the regional, provincial and local levels. Conceptually, the law defines disaster risk management (DRM) as the:

… continual process of social, professional, technical and scientific character for the formulation, execution, monitoring and assessment of policies, plans, programmes, regulations, instruments, standards, measures and permanent actions for knowing and reducing disaster risks, with the purpose of avoiding the generation of new disaster risks, reducing the existing ones and managing residual risk. (BCN 2021:Art. 2)

From this overly technical, abstract and comprehensive conceptualisation, the law establishes actions throughout the pervasive disaster management cycle (Bosher, Chmutina & Van Niekerk 2021). As such, the law defines four phases: mitigation (reducing existing risks and avoiding creating new ones); preparation (actions related to prevention and warning); response (in time of emergency per se, aiming to save lives and reducing impacts); and recovery (including rehabilitation and reconstruction, aiming to reestablish ‘normal conditions’) (BCN 2021:Art. 3). The law defines DRR as ‘activities aimed at preventing new disaster risks, reducing existing disaster risks and managing residual risk, all of which contribute to the country’s sustainable development’ (BCN 2021:Art. 2). Institutional coordination is conducted through ad hoc committees depending on the government level, and the different stages of the disaster management cycle, and critically, the level of emergency. The law offers an event-focused definition of emergency and establishes different levels (minor, major, disastrous and catastrophic) depending on the area impacted as well as the number of people and the administrative levels affected (BCN 2021:Art. 2). This new law is undoubtedly an advancement for Chile, not only in comparison to the previous ONEMI, but also in understanding the social character of disaster risks as influenced by the SFDRR. This new disaster law includes a more complex assessment of risks and promotes institutional coordination. As we show in the next section, the law has also created a new set of governance tools at different levels.

## Policy implementation at the subnational level

The new disaster law demands the elaboration of a number of emergency-related initiatives at the regional and provincial level, such as Emergency Plans, which are focused on response. However, it has also introduced new roles, competencies and tools for DRR to the different tiers of government, including the 345 municipalities in Chile. This includes the participation of elected authorities and technical parties in ad hoc committees for risk management (the *Comités para la Gestión del Riesgo de Desastres* [COGRID]); the demand to create DRM units at the local level; the development of local hazard maps; and the development of two plans at the local level: the emergency and the DRR plans (BCN 2021:Art. 27, 28, 32). These new functions demand emergent administrative and governance capacities, as well as the professionalisation (i.e. the need for formal training and qualifications, the development of expert knowledge on the topic, etc.) of local teams towards strategic planning in DRR and emergency response.

Formally, the new law forces the 345 Chilean municipalities to reorient local policies and instruments and consider DRR activities. However, while the Law 21.364 did create a Programme at the national level with a special budget for DRM instruments (BCN 2021:Art. 41), there is no special budget for local governments to enact these new policies and plans (Vergara-Saavedra & Ejsmentewicz 2024). The created Programme can be used by the regional, provincial and municipal levels, leading in practice to public funds for which local governments have to apply to and compete with other agencies. Given Chile’s spatial inequality, along with a funding structure of municipalities that forces them to self-fund part of their basic functioning (Montecinos 2022), municipal governments have had to resort to their own local budgets to support these new DRR instruments. In the context of incomplete decentralisation (Contreras, Guevara & Vásquez 2024), this means that local DRR instrumentation ends up competing with other priorities of local governments – and is not very high on the list, since DRR rarely leads to direct electoral gains.

**FIGURE 1:**
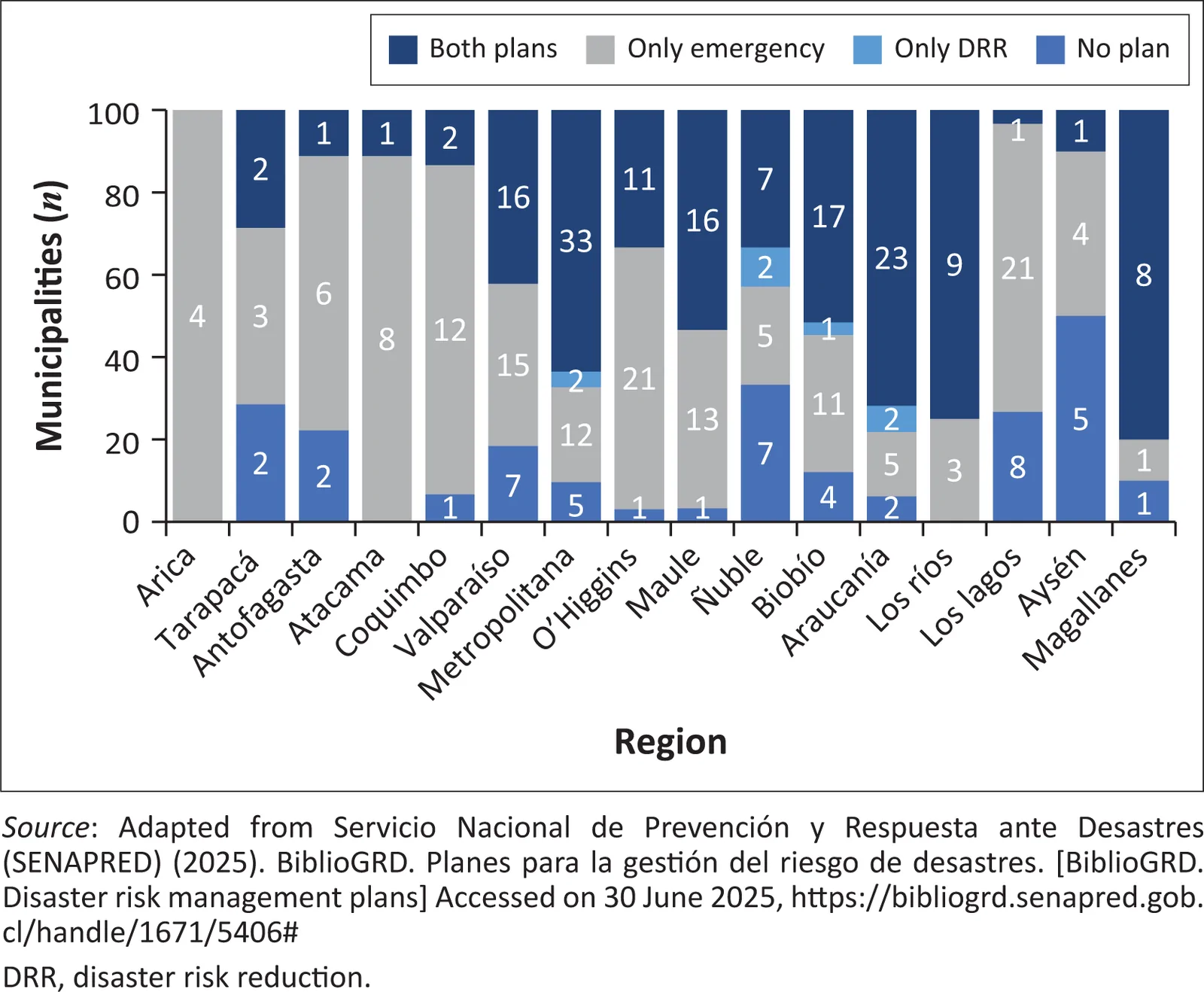
Implementation of Emergency and DRR plans at the local level in Chile, by region.

Despite limited funding, it is interesting to have an overview of the law’s local implementation. As mentioned, the law mandates municipalities to have an *Emergency Plan*, that is, the management tool that coordinates the actions and capacities during the response phase, which includes annexes by hazards (BCN 2021: Art. 32); and a *Disaster Risk Reduction Plan*, which orients and manages the set of actions, initiatives, projects and programmes considering the risks present in the local government’s jurisdiction (BCN 2001:Art. 28). *Servicio Nacional de Prevención y Respuesta ante Desastres* encourages the creation of these through detailed instructions and publicly available templates,^1^ as well as through the individual application of governments to their funding programme. As a result, data from June 2025 show that local government agencies are making progress with both plans. As shown in Figure 1, the municipalities with no plans are the minority throughout the regions (except for Aysen). Furthermore, 87% of local municipalities have at least one plan; 82% of which have an emergency plan, and 40% have a DRR plan. Therefore, the law is, at least formally, influencing local DRR policies.

In practice, however, the plans show important deficiencies in delineating concrete activities with municipal funds, are scantly including communities and are still widely focused on emergencies (Baeza 2025; Silva Bustos 2024). When analysed side by side, the plans are rather generic instruments that tend to look the same and have similar content. This is the expression of how the plans are being developed in practice: frequently, a private contractor funded through SENAPRED’s Programme is hired to develop a number of local DRR and emergency plans in one particular region or territory. The advancement of the plans is, ultimately, the accomplishment of legal demands in the context of the new disaster law and not a politically driven initiative to reduce local risks effectively.

The advancement of DRR and emergency plans can be complemented by considering another dimension of the new legislation at the municipal level, as the new disaster law also mandates the creation of units dedicated to DRM (BCN 2021:Art. 45). These units, which have the function of supporting and coordinating DRM in local governments, as well as elaborating both plans, are created by local mayors, and thus, also supported by municipal budgets. A study focused on the law’s implementation in the O’Higgins region,^2^ in the centre-south of Chile, shows the advancement of the creation of DRM units, where 70% of the 33 municipalities have a structure dedicated to this (either called DRM office, department, units or directions). It is telling, however, that some of these are created or nested within units dedicated to other functions, including public security (i.e. functions related to crime governance), civil protection, and even public works; hence further narrowing down the scope of DRR to a secluded subfield within another governance portfolio. Such fragmentation takes DRR away from addressing the root causes of vulnerability grounded in people’s everyday lives. Moreover, given that municipal officials frequently share responsibilities with other departments, the same study shows that only 48% of municipalities have personnel dedicated solely to these DRM units, and furthermore, most only have one person working. Finally, the study shows that people in charge of DRM offices lack relevant professional qualifications: while there are safety engineers, sociologists or social workers, many others are business managers, construction experts, audiovisual communicators or accountants.

When looking at the formal implementation of the Chilean disaster policy, it seems that there are some advancements at the local level. There are new functions instrumentalised in tools, municipal structures and personnel. In practice, however, at the municipal level, the law operates more as a symbolic formality – an isolated pocket of DRR activity, sometimes of secondary importance and nested within other governance portfolios. The development of plans describes an ineffective advancement, as these are being created externally to the municipality just to comply with the law. Regarding the embedded character of DRM units, this shows less integration and more of a lack of interest in developing a proper DRR. For all these reasons, the advancements so far are rather shallow and solely to conform to the formalities of the new law and its instrumentation.

## Conclusion

Ultimately, the Law 21.364 and the creation of SENAPRED reflect the institutionalisation of DRR in Chilean subnational governance structures. The law has created decentralised units, tools and personnel. Surely, in many cases, local governments have prioritised disaster-related issues and gained important capacities to prevent and respond to disasters. The law and its implementation, however, mirror a rather ambivalent progress. There is a tension regarding formal and actual capacities towards DRR, as it forces local structures to create and manage new functions. As such, the creation of planning tools may be rather useless, trapping disaster risk issues into a silo. While this institutional apparatus represents a successful story of DRR from the perspective of the SFDRR, it nonetheless maintains a focus on emergencies and fails to engage with the root causes of risk creation.

As mentioned, the state creates a particular way of enacting its objectives, which includes certain practices that structure ways of measuring and acting upon the world and that produce particular results. Hence, there is a particular conceptualisation and instrumentation behind a policy such as the one advanced by SENAPRED: disasters remain technical issues, and nature an outside and controllable matter. It is often the alleged *extra*-ordinary nature of natural hazards that justifies the call for more dedicated funding; hazards that sit outside of the everyday social fabric and that hence require a standalone machinery to deal with them. This is a throwback in disguise to the civil defence, military-devised paradigm of the extreme that we thought was long gone, but now raises its head again as climate change leads to increased concerns about so-called ‘extreme events’ and margination of everyday risks.

It is our contention that it is hence time to go beyond such siloing of DRR and move towards resisting risk creation (Wisner & Lavell 2017). Indeed, preventing disaster risk creation neither relies upon dedicated institutions nor designated funding. Rather, reducing existing disaster risk and preventing the creation of new ones entails redressing the uneven distribution of power and resources in society to the benefit of people whose voices are usually unheard and who prove most vulnerable. The responsibility for such an agenda falls on multiple and diverse government agencies, international organisations, NGOs and responsible private companies all together. This is thus a matter of political commitment to reducing inequalities – a task of everyday that extends much beyond specific and ad hoc DRR initiatives designed by dedicated agencies.

Clearly, such an approach to DRR does not suggest that specific funding and designated institutions are useless. It is indeed helpful when requested by local actors to address their own concerns, especially challenges that people face in their daily lives. Our point is rather that the absence of dedicated institutions and funding should not be used as an excuse to evade responsibility in slacking when it comes to addressing the root causes of disaster risk. Indeed, our contention is that one can do much with few specific resources. In fact, calling for designated agencies and dedicated funding may further enclose DRR within its own silo. On the contrary, everyday investments in economic development, housing, education, health, social services, arts and culture and environmental stewardship have a much more significant impact towards strengthening people’s everyday livelihood and well-being and, therefore, reducing existing risks and preventing the creation of new ones.

## Footnotes

1 See BiblioGRD section in the SENAPRED webpage, viewed 06 August 2025, from https://bibliogrd.senapred.gob.cl/handle/123456789/3363.

2 Unpublished report ‘Informe diagnóstico: Gobernanza del riesgo de desastre en comunas de la región del Libertador Bernardo O’Higgins’, developed as part of the SIA Project 85240326 funded by ANID. It focused on this region for the recent occurrence of disasters, with relevant floods occurring in June and August 2023. The aim of this study was to characterize the municipal units associated to DRM functions in the region, through assessing the 33 municipalities’ organization charts, human capital and installed capacities. The main sources were municipal web pages and transparency records.
